# Multilobar Insular-Involving Gliomas: Results with Hyperaggressive Resection

**DOI:** 10.7759/cureus.1623

**Published:** 2017-08-28

**Authors:** Michael Sughrue, Phillip A Bonney, Joshua D Burks, Jad Othman, Cordell Baker, Chad A Glenn, Charles Teo

**Affiliations:** 1 Department of Neurosurgery, University of Oklahoma Health Sciences Center; 2 Prince of Wales Private Hospital, University of New South Wales; 3 Neurosurgery, University of Oklahoma Health Sciences Center

**Keywords:** glioma, glioblastoma, insula, surgery, hyperaggressive, extent of resection, outcomes, survival, resection

## Abstract

Objective

Hyperaggressive resection refers to a philosophy that maximal resection should be pursued in gliomas, wherever possible. In this study, we provide a detailed report of the outcomes with hyperaggressive surgery for multilobar insular-involving gliomas (MIGs).

Methods

We report outcomes in patients with MIGs undergoing surgery aiming at gross total resection in all cases. Risk factors for neurologic deficits and survival were modeled using logistic and Cox regression.

Results

There were 72 consecutive patients, of whom 53 (74%) had undergone previous surgery. A greater than 90% resection was obtained in 67 patients (93%). Nineteen of 23 patients (83%) with Grade 2 tumors survived to the end of the follow-up period. Patients with Grade 3 tumors experienced 75% two-year survival rates and 48% four-year survival rates. Patients with Grade 4 tumors experienced 55% one-year survival rates and 33% two-year survival rates; eight of 33 patients (24%) lived longer than three years and three of 33 patients were alive at five years. Fifty-eight of 68 patients (85%) surviving to the three-month follow-up had a Karnofsky performance status (KPS) of 70 or greater, and 31 of 72 patients (43%) experienced improvement in KPS postoperatively. Permanent weakness occurred in 12 patients (17%), and permanent speech problems in three patients (13% of left-sided tumors).

Conclusion

Hyperaggressive surgical resection of MIGs yields rates of neurologic deficits within acceptable ranges and are lower than expected. In many cases, patients exceed the long-term survival expectations of conventional treatment.

## Introduction

While surgical resection of infiltrating gliomas is rarely curative, it is increasingly clear that optimal therapy involves the removal of as much of the tumor as possible. Complete removal is more readily accomplished in lower-risk regions, but uncertainty exists in how aggressive to be with tumors located in and near more central cerebral regions (opercular cortex, insula, basal ganglia, thalamus, and so on). Currently, many would agree that the standard of therapy consists of surgical resection with adjuvant radiotherapy and temozolomide.

Since Yasargil’s pioneering work, many have demonstrated that the insula can be removed with favorable morbidity in experienced hands [1-5]. The difficulty with extrapolating these experiences to clinical practice is that gliomas seldom involve the insula alone; instead, they more commonly invade other cerebral lobes and/or deeper structures. We term these more extensive tumors multilobar insular-involving gliomas (MIGs), to distinguish them from insula-limited tumors. To date, there has been a minimal effort to define the boundaries of very aggressive removal of these tumors using modern neurosurgical techniques. Currently, many would agree that the standard of therapy for glioma treatment consists of surgical resection followed by adjuvant radiotherapy and temozolomide. However, specific guidelines for treating MIGs are less clear.

Hyperaggressive resection refers to a philosophy that, given that a greater extent of resection improves survival in gliomas [4, 6-11], and that many neurologic deficits improve or resolve over time [4], the boundaries of resection should be pushed as much as possible. More specifically, hyperaggressive resection is the intention of resecting all of the tumor in all involved lobes. We have found that many patients will risk neurologic deficits to live longer, even when presented with the relative risks of aggressive resections. Consequently, we have approached MIGs with a 95%+ resection goal in all cases, stopping resection only to limit surgery directly in the internal capsule, motor cortex, or presumed speech areas.

Here, we provide the first detailed report regarding the outcomes of hyperaggressive surgery for MIGs in modern neurosurgery. We use imaging and regression analysis to determine which factors independently impact risk and survival benefit and, from this, we construct a tool to guide the preoperative risk-benefit considerations of a hyperaggressive approach relative to a more straightforward case.

## Materials and methods

Patient population

**Figure 1:**
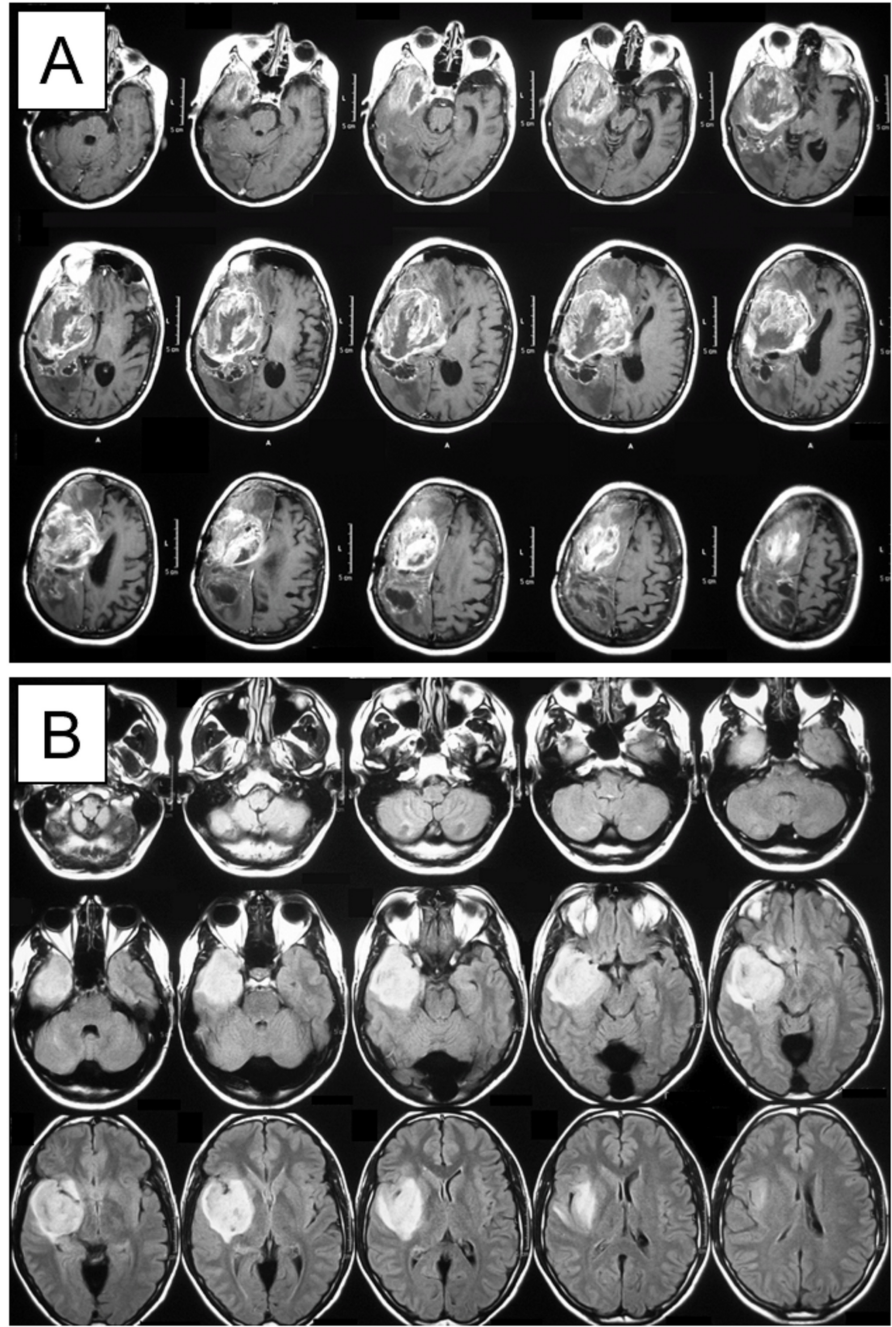
Examples of two MIGs used in the study Magnetic resonance imaging (MRI) of (A) contrast-enhancing tumor on T1-weighted sequence and (B) hyperintense tumor on T2/ Fluid Attenuated Inversion Recovery (FLAIR) sequence Multilobar insular-involving gliomas: MIGs

This study is a retrospective review of all insular glioma cases using a hyperaggressive approach at our center between 1995 and 2009. We define MIG as an infiltrating glioma involving at least one-third of the volume of the insula, with significant involvement of at least one additional adjacent cerebral lobe (frontal, temporal, or parietal). Two examples are provided in Figure 1, demonstrating the size and complexity of these tumors.

All patients underwent postoperative magnetic resonance imaging (MRI) scans in the first 48 hours, and thereafter as needed for oncologic management. The extent of resection (EOR) was evaluated on the first postoperative imaging using computerized volumetric analysis. This study was performed with the approval of and following local institutional human research guidelines.

Surgical technique and perioperative management

All surgeries were performed using minimally invasive keyhole techniques tailored to the exact pathoanatomic configuration of the patient’s tumor. Navigation was used in all cases. A holistic description of planning keyhole approaches to these tumors is beyond the scope of this paper. In short, we attempt to expose as little brain as possible and strive to adhere to recognized workhorse approaches. We generally avoid exposing any uninvolved opercular cortices and work through the transsylvian approach, when possible, to avoid disturbing normal insular cortices. Temporal - and/or parietal-predominant tumors are approached from a targeted anterolateral approach. Frontal-predominant tumors can be resected through a supraorbital eyebrow craniotomy or a frontally based anterolateral approach. All involved vessels are skeletonized and as much tumor as possible is removed from them. Resection proceeds until all tumor is removed or we encounter the internal capsule, presumed motor region, or presumed speech areas.

Postoperatively, steroids are weaned in two to five days, as tolerated. Patients with neurologic deficits are immediately started on aggressive physical, occupational, and/or speech therapy as an inpatient or outpatient. Patients without a history of seizures are not treated with antiepileptic therapy.

Data collection

For purposes of risk assessment and prognosis analysis, all preoperative imaging studies were analyzed to determine the involvement of the surrounding cerebral lobes and various cerebral structures, including the uncinate fasciculus, head of caudate nucleus, putamen/globus pallidus, internal capsule, thalamus, corpus callosum, and encasement of lenticulostriate arteries. For purposes of analysis, a frontal/temporal/parietal-predominant tumor was defined as a tumor with >50% of its volume in the relevant lobe. Importantly, this analysis distinguished between tumor involvement of the relevant structure and tumor involvement of the expected area of that structure, the structure having been displaced medially.

EOR was calculated using volumetric assessments, in which the total planimetric tumor volume on all slices was first calculated as a percentage of the total volume, to normalize values between imaging studies stored using different imaging techniques. EOR for largely non-enhancing tumors was calculated by a comparison of pre- and postoperative T2 imaging changes. EOR for enhancing tumors was calculated by comparing pre- and postoperative volumes of enhancement. Tumor and total brain volumes for a given image were analyzed using an identical scale, brightness, and contrast scanning, and were calculated for analogous brain regions between imaging studies. Tumor and brain outlines were traced by a neurosurgeon using Adobe Photoshop, and overlays of the tracings were exported and quantitatively assessed using the National Institute of Health’s (NIH's) ImageJ program.

Neurologic deficits were compared to the preoperative baseline, and the worsening of a pre-existing deficit or the presence of a new neurologic deficit were both considered neurologic complications. Neurologic deficits were considered permanent if persistent past six months postoperatively or until the last follow-up visit, whichever came first. Surgical complications included cerebrospinal fluid (CSF) leak, meningitis, wound infection, or breakdown. A central pathology review was performed by a board-certified pathologist using World Health Organization (WHO) guidelines [12]. Margins surrounding the resection were not analyzed, as this is not the standard at our institution. Clinical data were collected from patient records and telephone interviews. All clinical assessments were performed by a neurosurgeon.

Statistical analysis

Relationships between patient demographics were assessed using univariate analyses to identify potential between-group differences that might impact the rates of tumor recurrence. Binary variables were compared using Pearson’s χ2 test. Continuous variables were compared using an independent samples t-test or Analysis of Variance (ANOVA) after demonstrating the normality of data.

We performed logistic regression to determine the risk of postoperative speech and motor deficits. We first performed univariate analyses to determine variables associated with the increased risk of these deficits. Variables impacting the risk of these deficits with a p ≤ 0.2 on univariate analysis were included in stepwise binary logistic regression modeling [13]. We used a p value of 0.2 as this number is commonly used in screening for inclusion criteria. All odds ratios on multivariate analyses reflect the risk of having motor or speech problems at any time after surgery. The goodness of fit of the regression model was confirmed by demonstrating a nonsignificant p-value on the Hosmer-Lemeshow test [13-14].

Univariate analyses of factors impacting postoperative survival were performed using the Kaplan-Meier method, with the log-rank test used to compare survival between groups. All variables impacting survival in the univariate analyses with p ≤ 0.2 were included in regression modeling using stepwise Cox regression to calculate proportional hazard ratios.

For each regression model, we also tested interaction terms between each of the variables. The statistical significance of the interactions was assessed with backward conditional stepwise regression, estimating statistical significance by the likelihood-ratio test assessing the effect of removing interaction terms for all strata of the given variable [13]. After finding that none of the interaction terms would significantly alter the log likelihood of the regression model if removed (unadjusted p > 0.2 for all terms), we calculated adjusted hazard ratios without adjusting for interactions. 

Continuous variables are presented as a mean ± standard error. Standard error was used, as it is more representative of uncertainty. Statistical tests were considered significant when p < 0.05 after correcting for multiple comparisons.

## Results

Patient population

A total of 72 consecutive patients underwent hyperaggressive resection for MIGs at our center from 1995 to 2009. Basic demographic characteristics are listed in Table 1. This cohort included 33 glioblastomas, 16 WHO Grade III tumors, and 23 WHO Grade II tumors. Fifty-three patients (74%) underwent previous attempts at resection at another institution prior to our surgery, including 17/23 Grade II tumors (74%), 10/16 Grade III tumors (63%), and 26/33 Grade IV tumors (79%). Twenty-eight patients previously received radiotherapy, and 23 previously received chemotherapy. No patients received immunotherapy. The mean age at surgery was 42 ± 1.5 years (range: 16-69 years). There were 23 left-sided and 49 right-sided tumors. Median skin-to-skin operative time was 210 minutes (range: 100-467 minutes). Fifty-six patients had a KPS scale of ≥70 at the time of surgery. Forty-one patients were discharged on or before postoperative Day 3, with five patients going home the day after the surgery. Only three patients remained in the hospital longer than 10 days postoperatively. Severe permanent postoperative deficits, including hemiparesis, hemiplegia, dysphasia, and global aphasia, occurred in 16/72 patients. 

**Table 1:** Patient demographics Abbreviations. M:male; F:female; Pre-op: preoperative; KPS: Karnofsky Performance Status; Post-op: postoperative; SE: standard error; L: left; R: right

	Grade 2	Grade 3	Grade 4	All
Number of patients	23	16	33	72
Age (mean ± SE)	42 ± 2.6	43 ± 3.5	42 ± 2.0	42 ± 1.4
Gender (M/F)	17/6	11/5	20/13	48/24
Side (L/R)	6/17	6/10	11/22	23/49
Tumor volume				
< 50cc	14 (61%)	12 (75%)	16 (48%)	58%
≥ 50cc	9 (39%)	4 (25%)	17 (52%)	42%
Pre-op motor deficit	26%	25%	39%	32%
Pre-op speech deficit	17%	0%	21%	15%
Previous surgery	74%	63%	79%	74%
Previous radiation	4%	25%	70%	39%
Previous chemotherapy	4%	25%	48%	29%
Pre-op KPS > 70	91%	88%	70%	81%
KPS improvement post-op	35%	63%	39%	43%
KPS decline post-op	0%	13%	12%	8%
Post-op KPS > 70 at 3 months	100%	88%	61%	85%

The median tumor volume was 31 cc (range: 5-280 cc). Twenty-five patients (35%) had tumor volumes > 50 cc, and 10 patients (14%) had tumor volumes >100 cc. The frequency of various preoperative imaging characteristics is noted in Table 2. Eleven patients (15%) had significant preoperative speech difficulty and 23 (32%) had significant preoperative motor weakness.

**Table 2:** Preoperative imaging findings

	Grade 2	Grade 3	Grade 4	All
Three or more lobes	39%	50%	39%	42%
Frontal predominance	48%	63%	52%	53%
Parietal predominance	17%	13%	12%	14%
Temporal predominance	35%	25%	36%	33%
Frontal involvement	48%	75%	52%	56%
Parietal involvement	17%	31%	30%	26%
Temporal involvement	78%	50%	67%	67%
Uncinate fasciculus	9%	38%	24%	22%
Speech areas	4%	0%	27%	14%
Caudate head	13%	25%	12%	15%
Putamen/globus pallidus	26%	25%	24%	25%
Lenticulostriate encasement	22%	13%	12%	15%
Thalamus	13%	19%	6%	11%
Corpus callosum	0%	6%	12%	7%
Internal capsule	9%	13%	9%	10%

Extent of resection

Greater than 90% volumetric resection was obtained in 67 patients (93%). Greater than 95% resection was obtained in 55 patients (76%), including complete radiographic resection in 38 (53%). Three patients (4%) received 80-89% resection, and two patients (3%) received less than 80% resection. Examples of pre- and postoperative imaging of patients who underwent MIG resection is provided in the materials and methods portion of this manuscript.

Surgical complications

There were no perioperative deaths. Two patients had CSF leaks requiring surgical repair. There were no wound infections or additional episodes of wound breakdown. No epidural or subdural hematomas were noted on postoperative imaging.

Neurologic morbidity

Table 1 summarizes the effects of surgery on KPS scores. At the three-month postoperative follow-up, 31/72 patients (43%) experienced improvement in KPS and six patients (8%) had significantly decreased KPS as compared to preoperative scores. Fifty-eight of 68 patients (85%) alive at the three-month follow-up had KPS ≥70.

Thirty-nine patients (54%) had no neurologic deficits after surgery. Some degree of temporary or permanent limb weakness or hemiplegia occurred in 27 patients (38%), which was permanent in 12 patients (17%). Of 23 patients with left-sided tumors, postoperative dysphasia occurred in six patients, which was permanent in three patients (13% of left-sided tumors). Complete global aphasia occurred in one patient, which partially improved on follow-up. Visual field cuts were noted in 11 patients (15%), permanent dysesthesia in three patients (4%), and other neurologic deficits (memory problems, frontal syndromes) in six patients (8%).

Tables 3 demonstrates the results of multivariate analyses estimating the risk of motor and speech deficits. Logistic regression modeling found that only frontal predominance (odds ratio (OR) 4.3, 95% confidence interval (CI) 1.4 - 13.8, p=0.013) and encasement of lenticulostriate arteries (OR 4.4, 95% CI 1.0 - 20.6, p=0.057) increased the risk of neurologic weakness after aggressive surgical resection. Temporal predominance in left-sided tumors significantly decreased the risk of dysphasia compared to nontemporal MIGs (OR 0.1, 95% CI 0.01 - 0.7, p=0.026).

**Table 3:** Predictors of surgical risk and survival on multivariate analysis Abbreviations. HR: harzard ratio; CI: confidence interval; GP: globus pallidus; KPS: Karnofsky Performance Status

	Factor	HR (95% CI)	P Value
Survival	Grade 4	8.1 (2.5 – 26.7)	.001
	Grade 3	5.7 (1.6 – 20.4)	.007
	Volume > 50cc	4.4 (2.1 – 9.4)	.0001
	GP/Putamen involvement	2.6 (1.1 – 5.7)	.023
	KPS >70 at 3 Months	0.3 (0.1 – 0.7)	.005
Weakness	Frontal involvement	4.3 (1.4 – 13.8)	.013
	Lenticulostriate encasement	4.4 (1.0 – 20.6)	.057
Speech	Temporal predominance	0.1 (0.01 – 0.7)	.026

Survival after hyperaggressive resection

**Figure 2:**
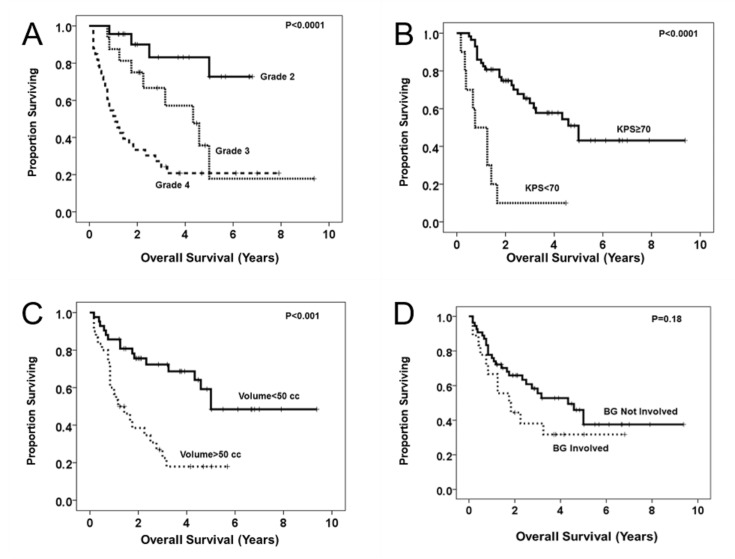
Kaplan-Meier survival plots of univariate predictors of postoperative survival Plots demonstrate survival stratified by histologic tumor grade (A), three-month postoperative Karnofsky Performance Status (KPS) scale (B), preoperative tumor volume (C), and invasion of basal ganglia (D).

Survival of patients by tumor grade is demonstrated in Figure 2A. There were four deaths from malignant transformation in Grade II tumors, with 19/23 Grade 2 patients surviving to the end of the follow-up period (83%). Patients with Grade III tumors experienced 75% two-year survival and 48% four-year survival. Patients with Grade IV tumors experienced 55% one-year survival and 33% two-year survival; eight of 33 patients (24%) lived longer than three years and three of 33 patients (9%) are alive at greater than five years of follow-up.

Figures 2B-D demonstrate Kaplan-Meier survival analyses for significant univariate predictors of survival in these patients. Table 4 additionally depicts the Cox-Regression analysis, modeling the hazard ratios (HR) for the survival of these variables. Not unexpectedly, histologic tumor grade and KPS < 70 at three months significantly impacted survival. Additionally, preoperative tumor volume > 50 cc (HR 4.4, 95% CI 2.1 - 9.4, p = 0.0001) or tumors invading the putamen and/or globus pallidus (OR 2.6, 95% CI 1.2 – 5.7, p = 0.023) independently conferred worse survival after controlling for tumor grade and KPS. There were few elderly patients in this series, and no matter how age was analyzed, it was not a significant predictor of mortality. We did not observe an extent-of-resection threshold for a survival benefit in this series, which is possibly explained by the similar extents of resection seen across the series. 

**Table 4:** Teo-Sughrue tool for preoperative risk-benefit assessment for MIGs Multilobar insular-involving gliomas: MIGs

Risk Factor	
Grade	
Grade 4	+ 8
Grade 3	+ 6
Grade 2	+ 0
Size	
Volume > 50 cc	+ 4
Volume < 50 cc	+ 0
Location	
Frontal predominance	+ 5
Parietal predominance	+ 1
Temporal predominance	+ 0
Involvement of deep structures	
Lenticulostriate encasement	+ 4
Globus pallidus/Putamen	+ 2
Suggested Interpretation	
Good Risk-Benefit	0 – 7
Moderate Risk-Benefit	8 – 14
Unfavorable Risk-Benefit	15 – 23

## Discussion

Decision making in glioma surgery inherently involves a value judgment balancing quality of life and length of survival. Many surgeons, knowing the generally dismal prognosis for this disease, take a conservative approach to avoid causing neurologic deficits in the face of the perceived modest benefit of surgery. We find that many patients, when offered the option and informed of the risks, choose living longer with the increased risk of a deficit over shorter survival without one. Further, unchecked tumor progression in these eloquent regions often eventually cause the same deficits these surgeons aim to avoid, which patients may not be informed about when the decision to pursue a conservative approach is made. The 32% rate of preoperative motor deficits in this series provides some insight into the natural history of these tumors. As we have demonstrated, neurologic deficits are not universal or even unacceptably common with hyperaggressive resection. Finally, many early deficits improve significantly or resolve, and some patients even improve after surgery.

In this study, we provide data on results obtained with hyperaggressive resection. Our data suggest that with this approach, neurologic deficits occur in fewer patients than may be expected, and those that do occur will frequently improve with time and aggressive rehabilitation. This suggests that with a good technique, the fear of neurologically devastating patients with these difficult tumors is often overstated. Specific areas of concern should be based on rigorous analyses of data gathered from operating on these patients using modern techniques—not on old teachings, anatomically-based hypotheses, or speculation.

Additionally, our data suggest that many patients may experience a survival benefit from aggressive resection. While this series is difficult to compare to others, given the selection criteria used in this study as well as the high rate of patients having received a prior operation, the fact that many of our recurrent glioblastoma patients live beyond one year after the second operation suggests that hyperaggressive surgery holds promise for these very ill patients. In patients with low-grade glioma, most remain in long-term remission years after their surgery, further suggesting that hyperaggressive surgery is at least an intriguing strategy and may merit consideration in these patients. This idea is supported further by the work of others. suggesting a survival benefit with the aggressive resection of these tumors [8, 11].

Risk-benefit assessment for MIGs

Surgical nihilism often leads to a self-fulfilling prophecy by creating outcomes with data that reinforce the nihilism [15]. Possibly due to this, there are insufficient data to determine the risk-benefit profile of attempting complete resection of MIGs. To address this gap in the literature, we have developed the Teo-Sughrue scale for a preoperative risk-benefit assessment of patients with MIGs, as demonstrated in Table 4. This scale was generated from the logistic and Cox regressions for motor and speech risk and postoperative survival, respectively. Given that covariates increasing the HR of death reduce the benefit (the risk/benefit ratio denominator) compared to tumors localized to the insula, all adverse variables found in the regression models worsen the risk/benefit ratio of hyperaggressive surgery. Thus, the risk ratios form an additive scale, where higher numbers imply a less favorable risk-benefit of removing the entire tumor.

A review of this scale reveals findings that are intuitive to experienced tumor surgeons. For instance, it is less fruitful to aggressively remove a glioblastoma than a Grade II astrocytoma and less fruitful to aggressively remove tumors when lenticulostriates are involved than when they are not involved. Of note, while the three-month postoperative KPS was an independent predictor of decreased survival, this was not predicted by preoperative KPS in this series; we frequently found that patients ultimately experienced improved KPS after resection. Thus, preoperative KPS was not included in this scale. It warrants mention that some judgment regarding KPS in preoperative decision making is necessary, and this represents a source of selection bias inherent in this study. We also acknowledge that it would be beneficial to analyze these results along with the O6-methylguanine-DNA-methyltransferase (MGMT) promoter methylation and the isocitrate dehydrogenase (IDH) mutant status of the tumors. Unfortunately, the majority of these data predate our realization of the significance of these genomic identifiers. 

Is a hyperaggressive approach worth the risk?

Clearly, it is still too early to conclude that greater than 90% resection in all MIG cases is an ideal goal. However, this study provides some modern-era insight of what a hyperaggressive strategy for these large, centrally located gliomas achieves. Certainly, the rates of neurologic deficits are somewhat higher with this strategy than one limiting aggressiveness through various adjuncts—what is interesting is that they are not drastically higher. Speech problems are permanent in only 4% of our patients, and motor deficits (while initially present in 38% of patients) resolve in over half of cases and the rate of permanent motor deficits is only slightly higher (17%) than that achieved with motor mapping in experienced hands [4]. 

## Conclusions

It is not possible to determine in a cohort of this size whether achieving greater than 90% resection in MIG cases conveys a survival benefit over a more conservative approach. Despite this, our data provide some suggestion that many patients exceed expectations of standard treatment, especially in light of the large percentage of patients undergoing repeat surgeries in this study. At best, we can conclude that some patients do very well with hyperaggressive resection, the risks are within reasonable levels given the fatal nature of this disease, and the decision of which is more important (length of survival vs. risk of deficit) should ultimately be made by the patient, accurately informed of all options by the surgeon.
